# Use of and confidence in YouTube and ChatGPT in surgical teaching and training

**DOI:** 10.1186/s12909-025-07992-0

**Published:** 2025-10-28

**Authors:** Stefan Tajsic, Imre Stjern-Vik, Dana Meknas, Martin Harbitz Bruusgaard

**Affiliations:** 1 Orthopaedic Department, University Hospital of North Norway, Tromsø, Norway; 2 Department of Community Medicine, Norwegian Centre for Rural Medicine, UiT The Arctic University of Norway, Tromsø, Norway; 3 Department of Clinical Medicine, UiT The Arctic University of Norway, Hansine Hansens Veg 18, Tromsø, 9037 Norway

**Keywords:** Surgical training, Medical education, YouTube, ChatGPT, Artificial intelligence

## Abstract

**Background:**

Today, YouTube and ChatGPT are used for a wide variety of purposes, ranging from entertainment to complex teaching. The content needs neither external approval nor quality assurance. We investigated how often doctors used YouTube and ChatGPT, how widespread their use was and the degree of confidence doctors had in these resources for teaching and training surgical procedures.

**Methods:**

In November 2023, we conducted a survey among doctors working in all the surgical departments of the University Hospital of North Norway, Tromsø. The questionnaire assessed use of and confidence in YouTube and ChatGPT and background data. For our analysis, we used frequency tables and association analysis. The study formed the basis for the second-year thesis of the first authors.

**Results:**

Out of a total of 152 doctors, 96 participated. Among the participants, 97% used YouTube and 14% used ChatGPT for teaching or training. There was no significant correlation between work experience and use (YouTube: *p* = 0.14, ChatGPT: *p* = 0.88). YouTube was used every month by 48%, while 2% used ChatGPT monthly. 60% of the doctors had a medium level of confidence that the content of YouTube was consistent with Norwegian procedures and standards, while 69% had low confidence in this aspect for ChatGPT.

**Conclusions:**

In November 2023, there was frequent and widespread use of YouTube to teach and train surgical procedures. The ChatGPT language model was rarely used, and the doctors had low confidence in its content. The future use of online learning platforms, language models and artificial intelligence for surgery training has an uncertain potential that warrants further research.

**Supplementary Information:**

The online version contains supplementary material available at 10.1186/s12909-025-07992-0.

## Background

YouTube is a video-sharing service founded in 2005 [1]. It is the largest video platform on the Internet and can be used for educational purposes [2]. A study of surgical trainees in various surgical departments in the UK showed that up to 87% routinely use online videos for learning, and that YouTube is one of the most frequently used platforms [3]. A cross-sectional study among medical students in the US states in its title: “YouTube is the most frequently used educational video source for surgical preparation” [4]. This is despite the fact that the content of YouTube is not peer-reviewed before publication [2].

Artificial intelligence (AI) has already gained a foothold in the healthcare sector [5]. It may be assumed that the use of AI and online learning videos will play a leading role in our future work as doctors [6]. The Chat Generative Pre-trained Transformer (ChatGPT) is an example of an AI tool that has increased the use of artificial intelligence since its launch in November 2022 [7]. It is becoming increasingly important to gain knowledge and insight into how doctors can use AI in their everyday clinical practice [8], particularly since AI is seen as a potential catalyst for the fourth industrial revolution [9]. As a form of information technology, AI can today perform tasks that normally require human intelligence [10]. New tools based on AI software are playing a key role in a variety of fields, including medicine [7, 11].

The aim of this study was to examine the frequency and extent of doctors’ use of YouTube and ChatGPT for the teaching and training of surgical procedures, in addition to their confidence in these two platforms for this purpose.

## Methods

The aim of this study was to examine the frequency and extent of doctors’ use of YouTube and ChatGPT for the teaching and training of surgical procedures, in addition to their confidence in these two platforms for this purpose. We conducted a cross-sectional study in November and December 2023. It was an exploratory study [12] with a sample consisting of consultants and specialist registrars at all ten surgical departments of the University Hospital of North Norway, Tromsø (UNN Tromsø).

### Questionnaire

We collected data with a non-validated questionnaire, which we had designed ourselves. The design was inspired by a previously published international study [4], and was piloted on one surgeon, one registrar and one general practitioner . The questionnaire was created on the online survey tool nettskjema.no The questions concerned the frequency and extent of the use of YouTube and ChatGPT for the teaching and training of surgical procedures, in addition to the doctor’s level of confidence that the content was consistent with Norwegian procedures and standards. See Appendix 1 for further details of the questions asked.

### Study population

The study population consisted of doctors from all surgical departments at UNN Tromsø who were present at the morning surgical meetings and understood written Norwegian. The heads of each department had agreed in advance to participation by their staff. At the morning meetings, the doctors were informed of the aim of the study and given a QR code for access to the questionnaire, which they completed on their own mobile phone or one provided by the researchers.

### Analysis

The statistical analyses were performed using the software Jamovi (version 2.4) and Microsoft Excel (version 16.81). Background variables were presented in frequency tables. Our null hypothesis was that there was no association between the use of YouTube or ChatGPT and length of work experience in terms of the different groups shown in Table 3. Fisher’s exact test was used to test the null hypothesis, using cross-tabulation (Table 3). This was due to larger tables than 2 × 2 and because several groups had an expected value < 5 (the Cochran criterion). The significance level was set at 5%.

## Results

A total of 96 out of 152 (63%) doctors working in all ten surgical departments of the hospital participated in the study.These consisted of 11 (12%) interns on surgical rotation, 35 (37%) residents specializing in a certain surgical specialty and 50 (52%) consultants. Table 1 presents the characteristics of the sample.

**Table 1:** Descriptive characteristics of the study population

Category	Response options	Frequency *n* (%)
Position	Interns on surgical rotation - level 1 (LIS 1)	11 (11.5)
	Residens specializing in a certain surgical specialty - level 2 (LIS 2)	12 (12.5)
	Residens specializing in a certain surgical specialty - level 3 (LIS 3)	23 (23.9)
	Consultant	50 (52.1)
Age (years)	24-30	18 (18.8)
	31-37	29 (30.2)
	38-44	22 (22.9)
	45-51	10 (10.4)
	52-58	7 (7.3)
	>58	10 (10.4)
Work experience (years)	0-4	36 (37.5)
	5-10	18 (18.8)
	11-20	24 (25.0)
	21-30	13 (13.5)
	>30	5 (5.2)

The findings revealed that 93 (97%) of the respondents had used YouTube for teaching and training purposes in the past year (Table 2). The figure for ChatGPT was much lower at 13 (14%). We found no statistically significant correlation between the frequency of use of the platforms and the length of work experience, *p* = 0.14 for YouTube and *p* = 0.88 for ChatGPT. Frequency of use of the platforms varied. YouTube was used every month by 48% percent, while 2% reported monthly use of ChatGPT, see Table 2.

**Table 2:** Use of and confidence in YouTube and ChatGPT

Category	Response options	Frequency *n* (%)
Use of YouTube	Never	3 (3.1)
	Once a year	11 (11.5)
	One or more times in six months	36 (37.5)
	One or more times a month	34 (35.4)
	Several times a week	12 (12.5)
	Every day	0
Confidence in YouTube	Low	28 (29.2)
	Medium	58 (60.4)
	High	10 (10.4)
Use of ChatGPT	Never	83 (86.5)
	Once a year	5 (5.2)
	One or more times in six months	6 (6.3)
	One or more times a month	2 (2.1)
	Several times a week	0
	Every day	0
Confidence in ChatGPT	Low	66 (68.8)
	Medium	30 (31.3)
	High	0
Future implementation of AI (years)	Never	1
	1-2	14 (14.6)
	3-5	35 (36.5)
	>5	34 (35.4)

Confidence that the content was consistent with Norwegian procedures and standards was medium for 60% of respondents regarding YouTube and low for 69% regarding ChatGPT, see Table 3.

**Table 3:** Use of YouTube and ChatGPT in relation to doctors’ work experience

Work experience (years)						
**Use of YouTube**	**0–4**	**5–10**	**11–20**	**21–30**	**> 30**	**Total**
Never	0	1	1	1	0	3
Once a year	1	2	2	4	2	11
One or more times in six months	14	6	10	4	2	36
One or more times a month	17	8	6	2	1	34
Several times a week	4	1	5	2	0	12
Every day	0	0	0	0	0	0
Total	36	18	24	13	5	96
**Use of ChatGPT**	**0–4**	**5–10**	**11–20**	**21–30**	**> 30**	**Total**
Never	31	15	20	12	5	83
Once a year	2	2	1	0	0	5
One or more times in six months	3	1	2	0	0	6
One or more times a month	0	0	1	1	0	2
Several times a week	0	0	0	0	0	0
Every day	0	0	0	0	0	0
Total	36	18	24	13	5	96

*P*-value using Fisher’s exact test for YouTube: p = 0.135

*P*-value using Fisher’s exact test for ChatGPT: *p* = 0.878

We also found that 35 (37%) of the doctors in the surgical departments believed that AI would be fully implemented in their practice within 3–5 years. By contrast, 12 (13%) doctors considered that it would never be realised in their practice (Table 2).

## Discussion

This cross-sectional study among surgeons at UNN Tromsø revealed that 97% had used YouTube and 14% ChatGPT for the teaching and training of surgical procedures. YouTube has existed longer than ChatGPT and the findings on its use concur with previous research [4]. Online learning videos have been shown to be useful for understanding complex three-dimensional concepts related to surgical procedures [4]. For example, in cardiac surgery, where spatial awareness is essential, the use of multimedia significantly improved student learning [13]. Our study shows that almost all the surgeons, regardless of work experience, used YouTube in their practice, which may be partly because surgery is a practical skill-based subject, where techniques are more effectively learned through visualisation [14]. Several lecturers at UiT the Arctic University of Norway even encourage medical students to watch YouTube as a supplement to the curriculum. However, the content of YouTube videos is of variable quality [2] and peer review or similar processes should be considered as a form of quality assurance [4]. Doctors who use YouTube must currently assess the content themselves and determine whether it complies with established national and international guidelines for surgical practice.

We believe that the low frequency of use of ChatGPT may be due to limited experience with this new service. In 2023, it was primarily a platform for the general public, answering people’s wide-ranging questions as if they were talking to another person [15]. Perhaps this indicates a greater barrier to the use of ChatGPT by surgeons for medical questions than its use by the general public? Initially, ChatGPT scored rather poorly (63%) on the United States Medical Licensing Examination (USMLE), but it has now improved and achieves a score of 90% [16]. The low score of ChatGPT in 2023 is probably part of the explanation for the low frequency of use found in our study. In 2024, the version of ChatGPT that scored 90% on the USMLE was offered free of charge [17], which suggests that a higher number of doctors using ChatGPT could be expected only one year after the study.

We found that the doctors had a low level of confidence in the content of ChatGPT. This may partly be explained by the findings of Au et al., who argue that ChatGPT has potential as an aid in surgical diagnosis and treatment but limitations in communication skills and understanding of human language [18]. A global study conducted in 2024 explored awareness and use of ChatGPT and other major language models among urologists. 59% of the participants felt that ChatGPT could not be used in clinical care due to the lack of validation [19]. Reliable references should therefore perhaps be used as a supplement to the text-generated answers provided by ChatGPT. This could rapidly increase confidence and popularity of ChatGPT [20]. A recent study among German-speaking orthopaedic surgeons shows that most expect AI to have a significant impact on orthopaedic surgery in the near future (5–10 years) [21].

UNN Tromsø has facilitated the integration of AI into everyday clinical practice. The Norwegian Centre for Clinical Artificial Intelligence is a collaboration between the UNN Health Trust, UiT The Arctic University of Norway and the Northern Norway Regional Health Authority [22]. One of its main tasks is “to facilitate and contribute to research, testing, innovation and implementation of AI in healthcare services to enhance everyday clinical care” [22]. Whether AI will be fully implemented in surgical practice within 3–5 years remains to be seen, but it is without doubt playing an ever greater role in specialist healthcare.

A strength of our study is the high response rate by surgeons. All surgical departments at UNN Tromsø participated in the study. Since we have studied the use of the platforms in a facility that is both a university hospital and a local hospital, where all the surgical departments were represented, we consider our results to be generalisable to similar hospitals in Norway. The population of the catchment area of UNN is much smaller than that of other hospitals in Norway. Could this be a factor in the doctors’ frequent use of YouTube?

The questionnaire we used was based on an exploratory design, which may have reduced validity and reliability [12]. In the first year following the launch of ChatGPT, we had already investigated how doctors in surgical departments at UNN Tromsø viewed the platform. This study therefore provides highly relevant insight into the potential role, development and use of ChatGPT among doctors in the years to come.

## Conclusion

Our findings indicate frequent and widespread use of YouTube by doctors to teach and train surgical procedures. In November 2023, the ChatGPT language model was used to a small extent, and the doctors had low confidence in its content. Our findings imply a clear need and potential to investigate further how online learning platforms, language models and AI are used in teaching and training in hospitals, primary care and medical degree programmes.

## Supplementary Information

Supplementary Material 1.

## Data Availability

The dataset can be made available on reasonable request to the main authors.
