# Supplementary material for: Use of and confidence in YouTube and ChatGPT in surgical teaching and training
Source: BMC Med Educ. 2025 Oct 28;25:1512. doi: 10.1186/s12909-025-07992-0 (PMC12570489; doi:10.1186/s12909-025-07992-0)
Supplement: Supplementary file 1 — Supplementary Material 1. [file 12909_2025_7992_MOESM1_ESM.pdf]

Appendix 1: Questionnaire provided to the doctors via the online survey tool nettskjema.no

Category Response options

Position Interns on surgical rotation - level 1 (LIS1)

Residence specializing in a certain surgical specialty - level 2 (LIS2)

Residence specializing in a certain surgical specialty - level 3 (LIS3)

Consultant

How old are you? (years)

How long have you worked in surgery? (years)

Have you used/do you use YouTube for the teaching, training and/or implementation of surgical procedures?

Never

Once a year

One or more times in six months

One or more times a month

Several times a week

Every day

How confident are you that the content of YouTube is consistent with Norwegian procedures and standards?

Low

Medium

High

Have you used/do you use ChatGPT for the teaching, training and/or implementation of surgical procedures?

Never

Once a year

One or more times in six months

One or more times a month

Several times a week

Every day

How confident are you that the content of ChatGPT is consistent with Norwegian procedures and standards?

Low

Medium

High

When will AI be fully implemented in your practice? (years)

Never

1-2

3-5

>5
